# Novel KDM6A splice-site mutation in kabuki syndrome with congenital hydrocephalus: a case report

**DOI:** 10.1186/s12881-018-0724-4

**Published:** 2018-12-03

**Authors:** Zhimei Guo, Fang Liu, Hai Jun Li

**Affiliations:** 1 Neonatology Department|, Bethune International Peace Hospital, Shijiazhuang, 050082 China; 2 Liver Disease Diagnosis and Treatment Center of PLA, Bethune International Peace Hospital, Shijiazhuang, 050082 China

**Keywords:** Kabuki syndrome, KDM6A, Congenital hydrocephalus; splice-site mutation

## Abstract

**Background:**

Kabuki syndrome (KS) is a rare congenital anomaly syndrome affecting multiple organs. Two genes have been shown to be mutated in patients with KS: lysine (K)-specific demethylase 6A (KDM6A) and lysine (K)-specific methyltransferase 2D (KMT2D, formerly MLL2). Although the congenital clinical characteristic is helpful in diagnosis of the KS, there are no reports of specific findings in fetuses that might suggest the syndrome prenatally.

**Case presentation:**

In this study, we described a male patient with a novel KDM6A splicing in exon(exon4) and flanking intron(intron3)-exon boundaries characterized by congenital hydrocephalus which had never been reported before. The male patient had inherited the c.335-1G > T splice site mutation from his mother who had fewer dysmorphic features than the patient who displayed a more severe phenotype with multiple organ involvement. Our research suggests that congenital hydrocephalus may accompany KS type 2, which improve the knowledge on KS further more.

**Conclusions:**

Based on genetic and clinical features, suggest that the c.335-1G > T splicing mutation in KDM6A causing KS-2 disease. At least for this case, we suggest that congenital hydrocephalus is closely associated with KS type 2.

**Electronic supplementary material:**

The online version of this article (10.1186/s12881-018-0724-4) contains supplementary material, which is available to authorized users.

## Background

Kabuki syndrome or Niikawa-Kuroki syndrome (KS) is a rare complex multi-system developmental disorder syndrome characterized by a stereotypical set of facial features, growth retardation, mild-to-moderate intellectual disability, organ malformation, hematological and endocrinological abnormalities [1–3], KS is also characterized by autoimmune disease and humoral immune deficiency [4]. Autosomal-dominant mutations in two epigenetic regulatory genes, lysine (K)-specific methyltransferase 2D (KMT2D/MLL2, OMIM 147920) and lysine demethylase 6A (KDM6A/UTX, OMIM 300867) cause KS, which led to the definition of two subtypes of KS: KMT2D-associated, autosomal-dominant KS type 1 (KS-1) and KDM6A-associated, X-linked-dominant KS type 2 (KS-2) [5–7]. Because of KS is a rare genetic syndrome, the vast majority of reported KS cases are sporadic. KS has an estimated incidence of 1 out of 32,000 live births [8]. However, this figure is likely to be underestimated due to missed diagnosis and misdiagnosis.

The use of the exome-sequencing strategy recently was able to identify KMT2D mutations as a major cause of KS, mutations in KMT2D were found in about 34–76% of KS patients [3, 5, 7, 9, 10]. In 2012, Lederer et al. reported 3 patients with KS who were negative for mutations in the KMT2D gene, they first identified complete or partial de novo deletions of KDM6A could cause KS [6], Miyake et al. first reported of KDM6A point mutations associated with KS-2 [11]. According to the literatures KDM6A mutations are detected in approximately 3 to 8% of patients with KS [7, 9]. In terms of mutation type, KMT2D and KDM6A show different profiles with regard to point mutations. Both genes show a large proportion of nonsense mutations and small deletions/insertions, but splice-site mutations are the most frequent mutation type for KDM6A as opposed to KMT2D where splice-site mutations play a minor role (27.5% vs. 7.9%) [7]. Three patients with KDM6A mutations displayed a severe developmental delay and intellectual disability with multiple organ involvement, but the female patient had mild phenotypic characteristics, this result suggested that the mutation type as well as X-inactivation pattern in affected organs in females may determine the severity of KS [10, 11]. Two brothers with KS-2 were identified a 4-bp deletion in the KDM6A gene, but their mother and maternal grandmother also carried the mutation and exhibited attenuated phenotypes, this report indicated that the first instance of hereditary X-linked KS [12].

In this study, we investigated a three-month-old boy and analysed his clinical manifestations and symptoms, with microcephaly, micrognathia, distinctive craniofacial features, high arched palate, postnatal growth deficiency, intellectual disability, dysplasia of hip joint and congenital hydrocephalus. By consulting the relevant database, we found that hydrocephalus phenotype was not included in the previous clinical characterization in OMIM. Based on genetic feature, a novel splicing mutation (c.335-1G > T) in KDM6A gene by Next-generation sequencing (NGS). Evidence of pathogenicity according to the ACMG, the mutation is splicing mutation, and no recorded in Normal public databases, and SPIDEX predict score is − 10.9497 (See the Additional file 1), so the splicing mutation is likely pathogenic. In the study provided us important information for early recognition and understanding of KS.

## Case presentation

We retrospectively collected the clinical records of the expectant mother from conception to birth. The general information and clinical features of the small male patient were recorded. The research was authorized by our hospital’s clinical ethics committee. Written informed consent document was signed from the patient’s family before data collection.

**Fig. 1:**
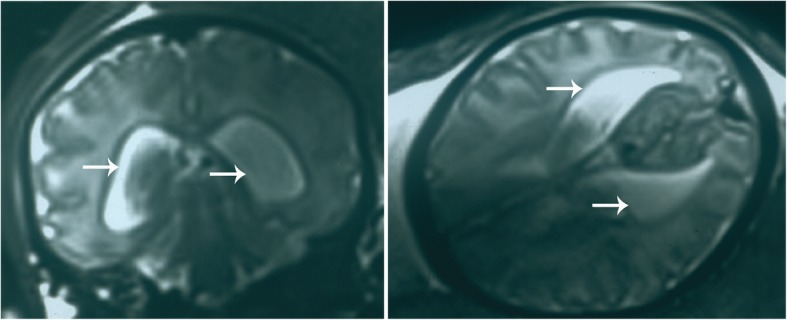
Head MRI, enlarged lateral ventricles indicated congenital hydrocephalus (white arrows)

A 2 months and 13 days old male was hospitalized in the neonatology department of our hospital for postnatal growth retardation on 20 February, 2017. The infant was spontaneous breech delivery at the 36th week plus 1 day of gestation with a weight of 2.8 kg and head circumference of 31 cm. The Apgar scores of the infant all were 8 at 1, 5, and 10 min. The infant was the third child of a 29-year-old mother who had fewer dysmorphic features and had two unaffected older sisters. Before the infant was born, magnetic resonance imaging (MRI) results showed that enlarged lateral ventricles (Fig. 1), this indicated congenital hydrocephalus.

**Fig. 2:**
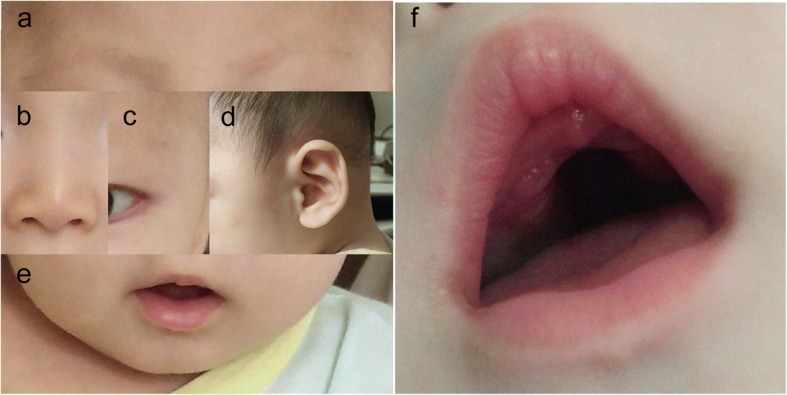
Typical patient abnormalitie. **a** Sparse eyebrows, **b** a depressed nasal tip, **c** long palpebral fissures with eversion of the lateral part of the lower eyelid, **d** large prominent ears with low set ears, **e** micrognathia, **f** gingival thickening and a high palate with cleft

**Fig. 3:**
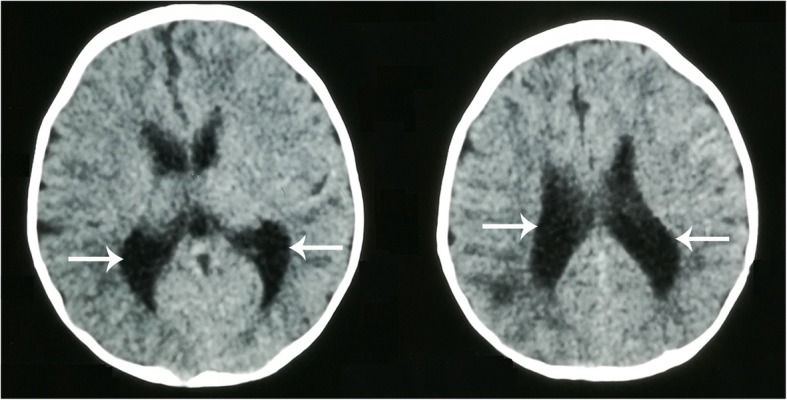
Head CT scan demonstrating enlarged lateral ventricles with hydrocephalus (white arrows)

**Fig. 4:**
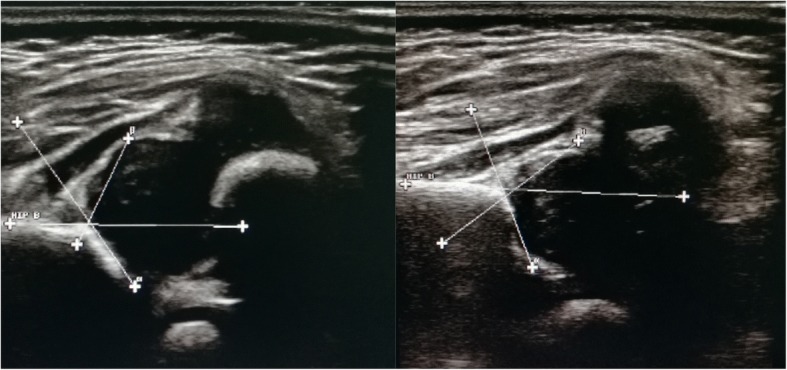
Ultrasonography showed developmental dysplasia of hip

After admission to our hospital, physical examination showed that the patient’s growth and development level was of below the normal range 3rd centiles at height (~ 52 cm), weight (~ 2.9 kg) and head circumference (~ 33.5 cm) in accordance with the new WHO (2006) Child growth standards [13], suggesting postnatal onset of growth retardation. The patient had an inability to lift the head and weak crying. The patient couldn’t amuse by physician, and couldn’t accomplish the tests of audio and visual tracking. However, The patient presented with recognizable facial features, including sparse eyebrows (Fig. 2a), a depressed nasal tip (Fig. 2b), long palpebral fissures with eversion of the lateral part of the lower eyelid (Fig. 2c), large prominent ears with low set ears (Fig. 2d), micrognathia (Fig. 2e), gingival thickening and a high palate with cleft (Fig. 2f), suggestive for KS. In addition, head computed tomography (CT) scan revealed hydrocephalus (Fig. 3), ultrasonography showed developmental dysplasia of hip (Fig. 4).

The results of cardiopulmonary and hepatolienal examination were normal. The patient had hypotonia, no pathological reflex. Liver and kidney functions, thyroid function, blood counts, electrolytes were normal. In addition, G-banding karyotyping showed that the patient had macroscopically normal chromosomes. Furthermore, heritage metabolic disease screening and antibody testing of maternal intrauterine infection were negative.

**Fig. 5:**
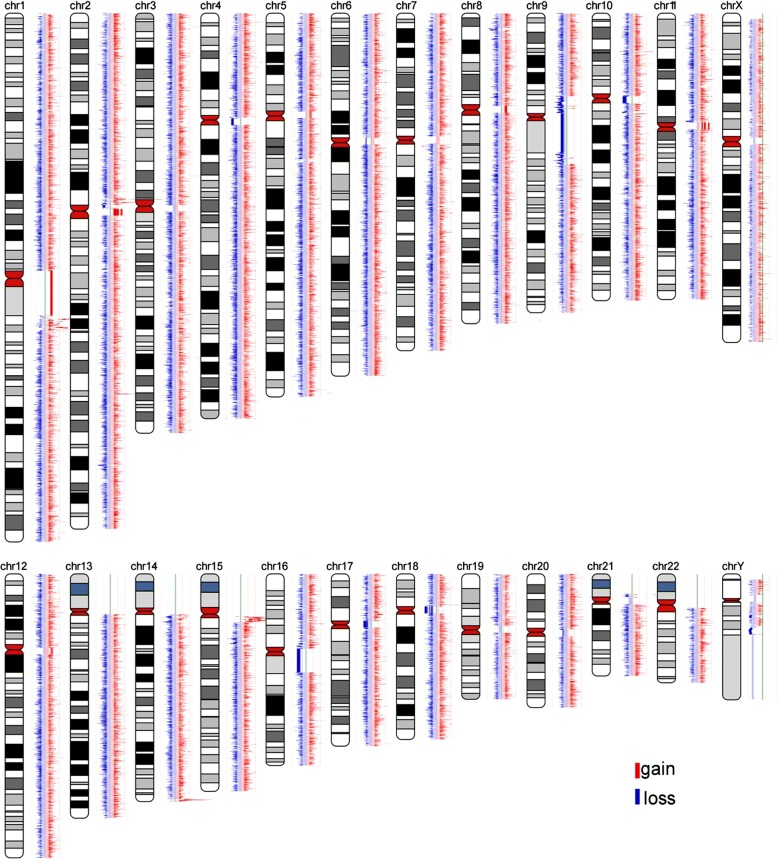
Whole-genome analyses of copy number variation of the patient

**Fig. 6:**
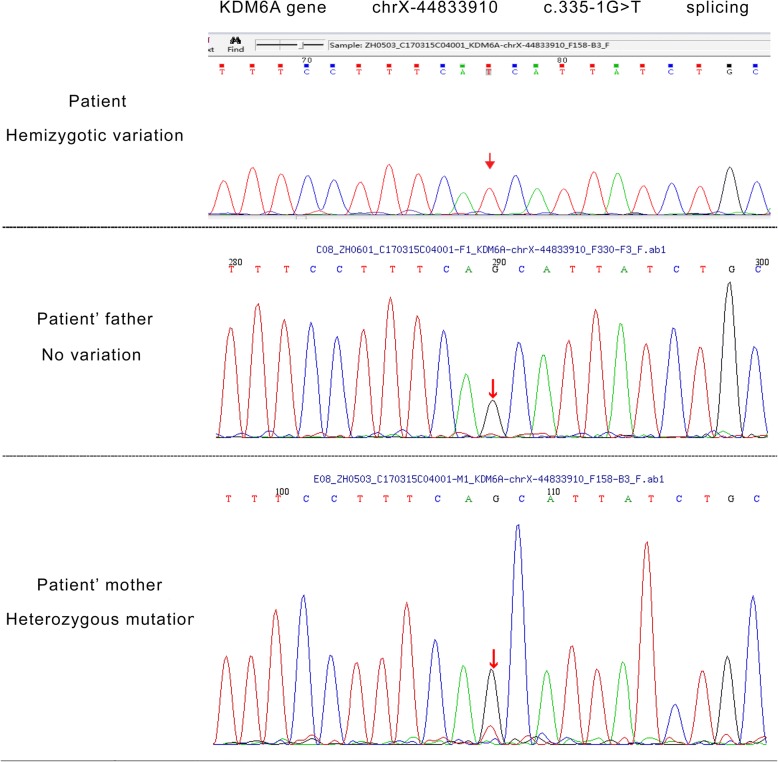
Sanger sequencing results for the patient and his mother and father

Due to the clinical manifestations, imaging and laboratory tests results, it was suspected that the patient may be suffering from KS. Genomic DNA was isolated from peripheral-blood leukocytes, whole-genome CNV was obtained by NGS following the manufacturer’s protocol (MyGenostics, Beijing, China). Whole-genome analyses of CNV have not found definite pathogenic gene (Fig. 5). All 19 coding exons of KDM6A and flanking intron-exon boundaries sequenced by medical exome sequencing and samples were sequenced on Illumina HiSeq Sequencer (Illumina, San Diego, CA). KDM6A gene was discovered c.335-1G > T splice site mutation in exon(exon4) and flanking intron(intron3)-exon boundaries. The significance of splicing mutations was estimated using the SPIDEX software. Sanger sequencing was performed in the patient to validate KDM6A gene mutation status, and the patient had inherited the c.335-1G > T splice-site mutation from his mother (Fig. 6).

## Discussion and conclusions

KS is a rare monogenic disorder that is characterized by characteristic facial features, postnatal growth retardation, intellectual disability, immunological dysfunction, endocrinological and hematological abnormalities, and organ malformation [5–7]. KS is a complex, heterogeneous disease and the manifestations of KS are various, a previous study had suggested high-arched eyebrows, short fifth fingers, and infantile hypotonia were less commonly seen in patients with KDM6A mutations than in those with KMT2D mutations. All of the patients with KDM6A mutations had short stature and postnatal growth retardation, only half of the patients with KMT2D mutations had these clinical features [11]. But recent study showed that the main clinical manifestations of KS were identified no specific for KS-1 or KS-2 which would allow distinguishing the two clinical subtypes [7]. In our study, the patient appears quite classical clinical facial features of KS, gingival thickening and a high palate with cleft, postnatal growth retardation, hypotonia, intellectual disability and developmental dysplasia of hip, but the most important characteristic of our patient is congenital hydrocephalus. Long et al. have reported two infants who presented with prenatal ascites, who were subsequently diagnosed with KS [14]. But before that, there are no distinct signs to suggest the syndrome prenatally, and there are no reports of specific findings in fetuses that might suggest the diagnosis. Kasuya et al. have reported for the first time, that the association of KS with hydrocephalus caused by aqueductal stenosis in an adult who was a 22-year-old at which this hydrocephalus was originally detected [15]. The association of KS with congenital hydrocephalus has not previously been reported in the literature and is presented in this case history for the first time. Although relatively non-specific, we suggest that congenital hydrocephalus in fetuses should be suspected to have KS.

Most of the published point mutations in KDM6A consist of nonsense mutations, small deletions, missense variants, and splice-site mutations. In addition, the published KDM6A mutations also included large deletions, large duplications/insertions and complex genomic rearrangement [7]. KDM6A gene was located on chromosome Xp11.3, KDM6A variants were detected in both male and female KS patients and could be shown to be de novo or inherited [11, 16]. KDM6A has been shown to escape X inactivation independently of the pseudoautosomal regions, suggesting a dosage contribution from both alleles in females [3]. Null expression of KDM6A in males and residual KDM6A expression from active X chromosomemay explain sex-biased severity [11]. However, in a previous study, the severity of clinical symptoms varied also among two female patients and a male with a KDM6A deletion [6]. Developmental delay and learning disability were generally moderate–severe in boys but mild–moderate in girls with KS-2 [17]. Furthermore, the KDM6A paralog on the Y chromosome, compensates for the reduced KDM6A transcript abundance in males [18]. Our research showed that the unaffected mother who also carried the mutation as had been reported patients with KDM6A deletions and mutations. This supported the view that KDM6A deletions and mutations, in both males and females, represented an X-linked dominant inheritance [12, 3]. We were able to confirm that female patients with KS-2 may have a rather mild manifestation of KS and may even develop normally with regard to cognitive function. The splice site mutation c.335-1G > T in KDM6A identified in the patient in our study had never been described in KS before.

In conclusion, we identified a novel splice site mutation of KDM6A in a Chinese boy with KS2. We consider his congenital hydrocephalus manifestations to be the character of KS and may be informative and valuable for prenatal diagnosis. This is the first report of a KDM6A splice site mutation in a Chinese patient, and further research is needed to confirm the finding. Our study demonstrated that the congenital clinical characteristic was helpful in diagnosis of the KS.

## Additional file

Additional file 1: Pathogenic evidence of the mutation in KDM6A gene. It shows the novel splicing mutation in KDM6A gene and the predication scores in various available predictive tools. We found PolyPhen-2, SIFT no predictiveed score, MutationTaster predictive was disease causing, GERP++_Predict is predicted Conserved result. SPIDEX predict score is − 10.9497, so the splicing mutation is likely pathogenic. (DOCX 15 kb)

## Abbreviations

CNV: Copy number variation
CT: Computed tomography
KDM6A: Lysine (K)-specific demethylase 6A
KMT2D: Lysine (K)-specific methyltransferase 2D
KS: Kabuki syndrome
MRI: Magnetic resonance imaging
NGS: Next Generation Sequencing
